# Towards genetic improvement of social behaviours in livestock using large-scale sensor data: data simulation and genetic analysis

**DOI:** 10.1186/s12711-023-00840-z

**Published:** 2023-09-28

**Authors:** Zhuoshi Wang, Harmen Doekes, Piter Bijma

**Affiliations:** Animal Breeding and Genomics, Wageningen University and Research, P.O. Box 338, 6700 AH Wageningen, The Netherlands

## Abstract

**Background:**

Harmful social behaviours, such as injurious feather pecking in poultry and tail biting in swine, reduce animal welfare and production efficiency. While these behaviours are heritable, selective breeding is still limited due to a lack of individual phenotyping methods for large groups and proper genetic models. In the near future, large-scale longitudinal data on social behaviours will become available, e.g. through computer vision techniques, and appropriate genetic models will be needed to analyse such data. In this paper, we investigated prospects for genetic improvement of social traits recorded in large groups by (1) developing models to simulate and analyse large-scale longitudinal data on social behaviours, and (2) investigating required sample sizes to obtain reasonable accuracies of estimated genetic parameters and breeding values (EBV).

**Results:**

Latent traits were defined as representing tendencies of individuals to be engaged in social interactions by distinguishing between performer and recipient effects. Animal movement was assumed random and without genetic variation, and performer and recipient interaction effects were assumed constant over time. Based on the literature, observed-scale heritabilities (${h}_{o}^{2}$) of performer and recipient effects were both set to 0.05, 0.1, or 0.2, and the genetic correlation (${r}_{A}$) between those effects was set to – 0.5, 0, or 0.5. Using agent-based modelling, we simulated ~ 200,000 interactions for 2000 animals (~ 1000 interactions per animal) with a half-sib family structure. Variance components and breeding values were estimated with a general linear mixed model. The estimated genetic parameters did not differ significantly from the true values. When all individuals and interactions were included in the analysis, the accuracy of EBV was 0.61, 0.70, and 0.76 for ${h}_{o}^{2}$ = 0.05, 0.1, and 0.2, respectively (for ${r}_{A}$= 0). Including 2000 individuals each with only ~ 100 interactions, already yielded promising accuracies of 0.47, 0.60, and 0.71 for ${h}_{o}^{2}$ = 0.05, 0.1, and 0.2, respectively (with ${r}_{A}$ = 0). Similar results were found with ${r}_{A}$ of – 0.5 or 0.5.

**Conclusions:**

We developed models to simulate and genetically analyse social behaviours for animals that are kept in large groups, anticipating the availability of large-scale longitudinal data in the near future. We obtained promising accuracies of EBV with ~ 100 interactions per individual, which would correspond to a few weeks of recording. Therefore, we conclude that animal breeding can be a promising strategy to improve social behaviours in livestock.

**Supplementary Information:**

The online version contains supplementary material available at 10.1186/s12711-023-00840-z.

## Background

Modern animal production systems, which are characterized by group-housing with more opportunity for animal movement and ample contacts between individuals, are believed to improve animal welfare. However, domestic animals regularly engage in social interactions, and the high demand for animal productivity has led to intensification of the pig/poultry industry. This intensification has brought new challenges for animal welfare and health, including an increase in harmful social interactions, such as injurious feather pecking in poultry and tail biting in swine.

To reduce the occurrence of harmful social behaviours, management strategies can be applied. Physical alteration of animals (e.g. beak trimming in poultry or tail docking in pigs) has been widely applied, but this has undesired implications for animal health and welfare [1] and has been banned in many countries. The use of smaller group sizes may also reduce harmful behaviours, as it reduces the number of (potential) interactions between animals [2], but most modern farms have relatively large groups. Alternative management solutions include providing appropriate rearing conditions to reduce feather pecking in laying hens [3, 4], using enriched housing to reduce tail biting in pigs [5], and increasing feeding space to reduce aggressive behaviours in dairy cows [6]. These husbandry solutions may improve or have already improved social behaviours in different species. However, many of them are prohibitively expensive, labour intensive, or otherwise difficult to incorporate into routine management. The limited efficacy or questionable ethical justification of current management solutions has stimulated the interest in the use of genetics to improve social behaviours, which has the potential to lead to cumulative and permanent benefits at relatively low costs for individual producers [7].

Over the past century, animal breeding has made a substantial contribution to improving the efficiency of animal production [8]. However, the contribution of animal breeding to the improvement of behavioural traits is still limited [9]. In a meta-analysis, Dochterman et al. [10] found an average heritability for behavioural traits of ~ 0.25, with most behaviours showing a heritability between 0.15 and 0.29. Kjaer and Sørensen [11] found heritabilities in the range from 0.05 to 0.20 for the number of pecks performed by laying hens, based on an average of ~ 25 pecks per individual. Agha et al. [12] found that the heritability estimate for behavioural traits derived from social network analysis in pigs ranged from 0.09 to 0.26. Desire et al. [13] estimated heritabilities ranging from 0.09 to 0.44 for skin lesions in pigs. These results indicate that genetic improvement of behaviours is possible, in principle. One challenge of applying breeding strategies for behaviour-related traits is that the phenotype of an individual may depend not only on the effect of the genes of the individual itself (known as the direct genetic effect, DGE), but also on the genetic effects of the genes of its group mates (known as social or indirect genetic effects, IGE) [14, 15]. Over the last two decades, social genetic models have been developed to quantify DGE and IGE and to optimize breeding schemes for traits related to social behaviours (e.g. [14, 16–19]). Results show that IGE are present for a wide range of traits and can have a substantial impact on heritable variation and response to selection. For example, in laying hens showing pecking behaviour, IGE contribute 33 to 76% of the heritable variation in survival time [20, 21]. IGE models are typically applied to individual traits that are affected by social interactions, such as growth rate, feed intake, and tail injuries in pigs, or mortality and feather condition due to pecking in laying hens, rather than to the social behaviour itself (e.g. [14]). Hence, these models are usually fitted to phenotypes of the recipient of the behaviour, and their application does not require data on the behavioural interactions that cause the IGE. Instead, the IGE (i.e. the genetic effects of the performers) are identified in a statistical manner, using information on group composition [22].

However, a limitation of these models is that they assume that each individual affects the phenotype of each of its pen mates equally. Therefore, they are particularly useful in the case of small groups, where animals have ample interactions with all their pen mates [23, 24]. However, for large groups, the assumption of equal interaction does not hold, e.g. because an individual can only interact with another individual when they are physically close enough. Thus, in large groups with hundreds or even thousands of individuals, one individual can only interact with the limited number of individuals in its homing range.

Radersma [25] presented a framework to estimate the social tendencies of individuals from the strength of undirected pairwise social interactions obtained from social network analysis. The probability of pairwise social interactions was modelled by taking the logit of the sum of the two social tendencies weighted by the tendencies’ social governance. The social governance represents how much an individual affects the social interaction frequency relative to other individuals. In this framework, social tendency is a normally distributed latent trait that explains the observed variation in the frequency of interactions between pairs of individuals. This social tendency consists of a heritable component (breeding value, or BV) and a random environmental component, similar to an ordinary quantitative genetic trait. Therefore, classic animal breeding theory also applies to social tendency.

Assuming a single social tendency, as in Radersma [26], is not always appropriate. Many behavioural interactions are directional and an individual can play one of two roles in an interaction event: performer or recipient. For a pair of animals, say A and B, the probability that A performs and B receives the behaviour is not necessarily equal to the probability that B performs and A receives the behaviour. Quantitative genetic studies on feather condition score in laying hens, for example, showed that the genetic correlation between direct and social effects is clearly different from 1 [27], suggesting that performer and recipient effects are two distinct quantitative genetic traits. Therefore, two distinct traits should be defined when individuals can play two distinct roles. Moreover, if we can clearly distinguish the performer and the recipient, then the term IGE in a classic social genetic model refers to the genetic effect of the performer, and DGE refers to the genetic effect of the recipient because in classic social genetic models, traits are usually measured on recipients.

Genetic analysis of phenotypes that are a result of pairwise social interactions requires extensive records of the social interactions, including not only the event itself but also the identity of the participants. Recording all interactions between individuals in a large population used to be time- and labor-demanding, but it is gradually becoming feasible due to recent developments in animal detection and tracking techniques. By combining a variety of sensors and artificial intelligence (AI) algorithms, researchers have successfully detected animal behaviour in several species [26, 28]. These technologies can provide big data on social interactions between individuals, and we expect that such data will become available in the coming years. However, there is still a lack of statistical genetic methods to translate such data into estimated breeding values (EBV) for social traits, and little is known of the sample sizes required for estimating genetic parameters and BV.

The objectives of this paper were to (1) extend the social genetic model of Radersma [26] for genetic analysis by including both performer and recipient effects, and (2) investigate required sample sizes by evaluating the effect of the number of individuals, the number of interaction records, and group size on accuracy and bias of estimated genetic parameters and BV. To address these objectives, we used simulation of animal behaviour in time and space, with subsequent genetic analysis of the resulting data. First, latent traits were defined to represent the tendency of individuals to be engaged in behavioural interactions by distinguishing between performer and recipient effects. Second, social interactions with known performer and recipient were simulated under various population settings based on an assumed genetic structure, and for different values of heritabilities, genetic variances, and the genetic correlation between performer and recipient effects. And third, statistical models were applied to estimate the relevant genetic parameters (genetic and environmental variances and correlations) and BV of the social trait from the simulated data, and to investigate the accuracies and bias of the EBV. Input values for the simulation were chosen such that the estimated observed-scale heritabilities corresponded to common values for behavioural traits found in the literature. We assumed that movement of animals was random and showed no genetic variation, and that performer and recipient effects were constant over time (see “Discussion” section).

## Methods

### Trait definition

For each individual, two latent traits were defined to represent its tendency to be engaged in a behavioural interaction as a performer (trait $\alpha$) or recipient (trait $\beta$). Each of the two traits consisted of two parts: a heritable part (the BV) and a permanent non-genetic effect (known as “permanent environment”). Thus, for the $i$th individual:

1 $${P}_{\alpha ,i}={\mu }_{\alpha }+{A}_{\alpha ,i}+{Ep}_{\alpha ,i},$$

2 $${P}_{\beta ,i}={\mu }_{\beta }+{A}_{\beta ,i}+{Ep}_{\beta ,i,}$$

where ${P}_{\alpha ,i}$ and ${P}_{\beta ,i}$ are the individual’s total tendency for each trait, ${\mu }_{\alpha }$ and ${\mu }_{\beta }$ are the means of ${P}_{\alpha }$ and ${P}_{\beta }$, ${A}_{\alpha ,i}$ and ${A}_{\beta ,i}$ are the individual random BV, and ${Ep}_{\alpha i}$ and ${Ep}_{\beta i}$ are the random permanent environmental effects. No residuals were simulated on the latent scale (see below). Random genetic effects of base generation individuals were sampled from a bivariate normal distribution,

$$\left[\begin{array}{c}{A}_{\alpha }\\ {A}_{\beta }\end{array}\right]\sim N\left[\left(\begin{array}{c}0\\ 0\end{array}\right),\left(\begin{array}{cc}{\sigma }_{{A}_{\alpha }}^{2}& {r}_{A}{\sigma }_{{A}_{\alpha }}{\sigma }_{{A}_{\beta }}\\ {r}_{A}{\sigma }_{{A}_{\alpha }}{\sigma }_{{A}_{\beta }}& {\sigma }_{{A}_{\beta }}^{2}\end{array}\right)\right],$$

where ${r}_{A}$ is the genetic correlation between traits $\alpha$ and $\beta$. For the next generation, we simulated a half-sib family structure. The BV of the offspring were generated as:

3 $${A}_{\alpha ,off\;spring}=\frac{1}{2}{A}_{\alpha ,sire}+\frac{1}{2}{A}_{\alpha ,dam}+{MS}_{\alpha ,off\;spring},$$

4 $${A}_{\beta ,off\;spring}=\frac{1}{2}{A}_{\beta ,sire}+\frac{1}{2}{A}_{\beta ,dam}+{MS}_{\beta ,off\;spring},$$

where $MS$ was the Mendelian sampling term, sampled from:

$$N\left[\left(\begin{array}{c}0\\ 0\end{array}\right),\frac{1}{2}\left(\begin{array}{cc}{\sigma }_{{A}_{\alpha }}^{2}& {r}_{A}{\sigma }_{{A}_{\alpha }}{\sigma }_{{A}_{\beta }}\\ {r}_{A}{\sigma }_{{A}_{\alpha }}{\sigma }_{{A}_{\beta }}& {\sigma }_{{A}_{\beta }}^{2}\end{array}\right)\right].$$

Random permanent environmental effects for the offspring generation were sampled from:

$$\left[\begin{array}{c}{Ep}_{\alpha }\\ {Ep}_{\beta }\end{array}\right]\sim N\left[\left(\begin{array}{c}0\\ 0\end{array}\right),\left(\begin{array}{cc}{\sigma }_{{E}_{{P}_{\alpha }}}^{2}& 0\\ 0& {\sigma }_{{E}_{{P}_{\beta }}}^{2}\end{array}\right)\right],$$

where the correlation between the permanent environmental effects of an individual on traits $\alpha$ and $\beta$ was assumed equal to zero.

Behavioural interactions were simulated for the offspring generation only. For an interaction between individuals $i$ and $j$ when they are physically close (see below for conditions related to their proximity), the probability of $i$ performing the social interaction towards $j$ is given by:

5 $${p}_{ij}=logistic\left({P}_{\alpha ,i}+{P}_{\beta ,j}\right)=\frac{1}{1+{e}^{-\left({P}_{\alpha ,i}+{P}_{\beta ,j}\right)}},$$

where $({P}_{\alpha ,i}+{P}_{\beta ,j})$ ∈ ℝ, is the normally distributed tendency that $i$ as performer and $j$ as recipient were engaged in a social interaction. The logistic function was used to rescale ${P}_{\alpha ,i}+{P}_{\beta ,j}$ from the real number domain to the probability domain ranging from 0 to 1. Binary interaction records were then generated by sampling a random number from a Bernoulli distribution with parameter ${p}_{ij}$, where 1 means that the interaction took place, while 0 means that it did not take place.

The total variance underlying the binary records is the result of five components: ${A}_{\alpha ,i}$, ${Ep}_{\alpha ,i}$, ${A}_{\beta ,j}$, ${Ep}_{\beta ,j}$, and the variance induced by the Bernoulli sampling process. The latter is known as the link variance [29] and is equal to $\frac{{\pi }^{2}}{3}$ for the logistic link function. Because this model is equivalent to a threshold model with a logistically distributed residual on the underlying scale of the latent variable, there is no need to include a residual in Eqs. (1) and (2) (see Dempster and Lerner [30] for the threshold model). Therefore, the heritability for the performer trait on the underlying scale is given by:

6 $${h}_{{u}_{\alpha }}^{2}=\frac{{\sigma }_{{A}_{\alpha }}^{2}}{{\sigma }_{{A}_{\alpha }}^{2}+{\sigma }_{{A}_{\beta }}^{2}+ {\sigma }_{{Ep}_{\alpha }}^{2}+{\sigma }_{{Ep}_{\beta }}^{2}+\frac{{\pi }^{2}}{3}}.$$

The analogous expression applies to the heritability for the recipient trait ($\beta$).

### Simulation of animal movement

For social interactions to occur, animals need to be in each other’s proximity. Thus, the physical position of the individuals over time in the pen must be simulated. We used an Agent Based Model [31] with three behaviours underlying individual movement: (1) eating, where the individual first moved to a target (a feeder that was located on the edge of the pen) and then stayed for a while until it was satisfied; (2) walking, where the individual moves into a random direction; and (3) resting, where the individual did not move at all. These behaviours and the resulting physical position of individuals were simulated in discrete time steps. For each individual and each time step $t$, the motivation for the three behaviours was defined as a motivation vector:

7 $${\mathbf{m}}_{i,t}=\left(\begin{array}{c}{M}_{Ei,t}\\ {M}_{Wi,t}\\ {M}_{Ri,t}\end{array}\right),$$

where ${M}_{Ei,t}$, ${M}_{Wi,t}$, and ${M}_{Ri,t}$ denote the individual’s motivation for eating, walking, and resting, respectively. The change of motivation in each time step is given by the matrix ${\varvec{\Delta}}$:

8 $${\Delta }_{\mathrm{t},\mathrm{t}+1}={\left(\begin{array}{c}\begin{array}{ccc}{\Delta }_{E\leftarrow E}& {\Delta }_{E\leftarrow W}& {\Delta }_{E\leftarrow R}\end{array}\\ \begin{array}{ccc}{\Delta }_{W\leftarrow E}& {\Delta }_{W\leftarrow W}& {\Delta }_{W\leftarrow R}\end{array}\\ \begin{array}{ccc}{\Delta }_{R\leftarrow E}& {\Delta }_{R\leftarrow W}& {\Delta }_{R\leftarrow R}\end{array}\end{array}\right)}_{t,t+1},$$

where ${\Delta }_{B\leftarrow B^{\prime}}, (B,B^{\prime} = E,W,R)$ is the change of motivation in behaviour $B$ resulting from the expression of behaviour $B^{\prime}$. Therefore, in the next time step, the new motivation vector is:

9 $${\mathbf{m}}_{i,t+1}={\mathbf{m}}_{i,t}+\left(\begin{array}{c}{\Delta }_{E\leftarrow B^{\prime}}\\ {\Delta }_{W\leftarrow B^{\prime}}\\ {\Delta }_{R\leftarrow B^{\prime}}\end{array}\right),$$

with $$B^{\prime}=E,W,R$$.

The elements of matrix $\Delta$ determine the timespan that individuals typically spend on a certain behaviour and the behaviour they express after that. The diagonal elements in matrix $\Delta$ were negative, meaning that the motivation for a behaviour decreased when an individual performed that specific behaviour. The off-diagonal elements were positive, meaning that when the individual was performing one behaviour, the motivation of the other two behaviours increased.

**Fig. 1:**
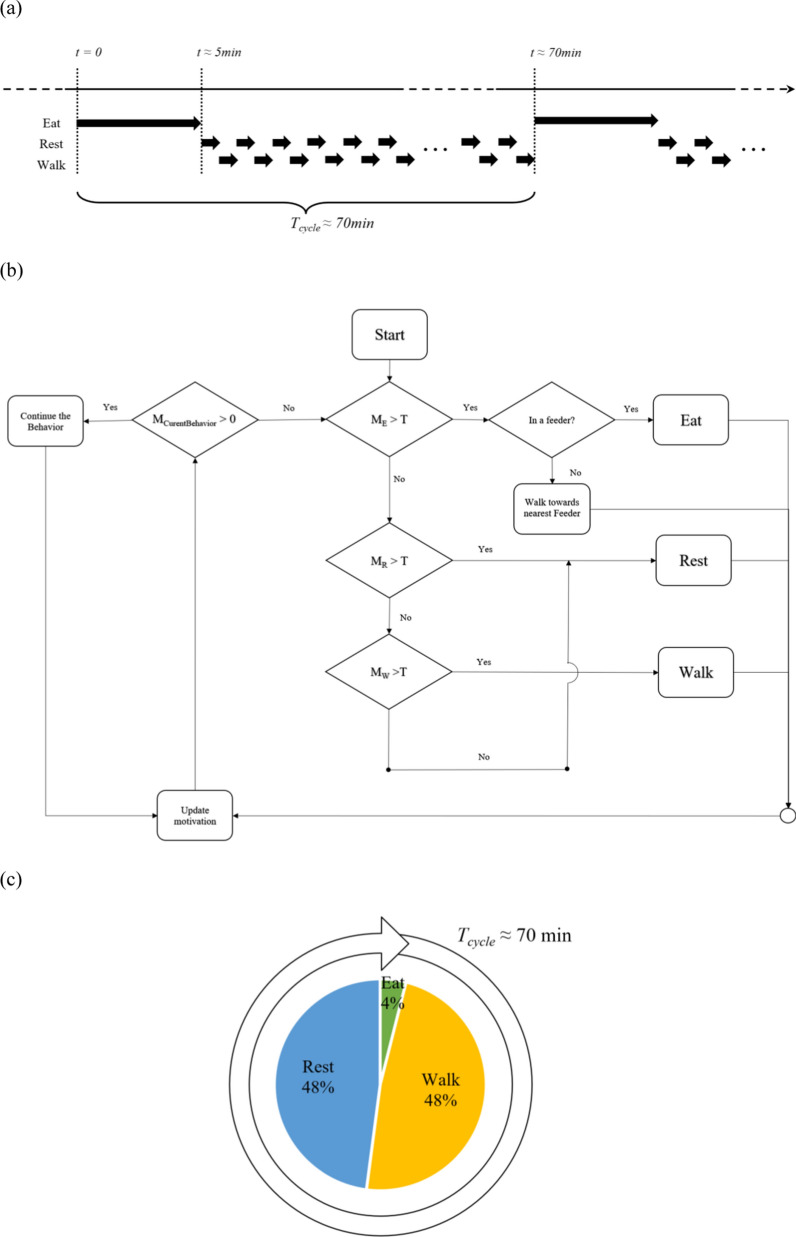
Simulated behavioural pattern and decision tree of agents. **a** Behavioural cycle. Individuals will visit the feeder approximately every 70 min. The meal lasted 5 min on average (including the time to approach a feeder). Between two visits of the feeder, the individuals would frequently switch between random walking and resting behaviour. The duration of walking and resting were both ~ 20 s. **b** Decision tree. Eating had the highest priority and walking had the lowest. At the beginning of each time step, the program would check if the motivation of the behaviour in the previous step was still above zero. If this was true, then the individual will continue the behaviour, without performing other behaviours even if the motivation of those behaviours was greater than the threshold value. This means that once the individual started a behaviour, it would not initiate a new behaviour until the motivation for the current behaviour fell below zero. When none of the behaviours exceeded the threshold an individual would rest. **c** Time budget. In the simulation, individuals on average go to the feeder every 70 min and spend 4% of the total time on eating. Walking and resting evenly take up the rest of the time

The individual starts a behaviour when the motivation was greater than a preset threshold $T$ ($T$ > 0) and terminates the behaviour when the motivation is zero. We chose a $T$ of 100, so that the motivation can approximately be interpreted as a percentage for how motivated the animal is for a behaviour. A motivation of 100 for e.g. eating behaviour, means that the animal moves to a feeder and starts eating (if a free feeder is available); the animal stops eating when the motivation reaches zero. The time interval between meals for each animal was set to ~ 70 min and the duration of each meal was set to ~ 3 min (Fig. 1a). The animal-to-feeder ratio was set to 5:1, which was sufficient for animals not having to wait too long to eat when they are motivated. For each individual, the initial motivations were uniformly, randomly, and independently chosen from the interval 0 to $T$, and the initial position in the pen was random as well. The elements of matrix $\Delta$ were chosen to fulfill the behavioural pattern shown in Fig. 1a, where an individual visits the feeder every ~ 70 min and, between two meals, frequently switches between walking and resting for details, (see Additional file 1: Text S1 and Fig. S1). Animals can be motivated for multiple behaviours at the same time. In these cases, eating was given the highest priority and walking the lowest priority, as defined in the behaviour decision tree (Fig. 1b). Figure 1c shows the resulting time budget of the animals for each behaviour.

The physical position of all animals in the pen was simulated at every time step using the approach described in Additional file 1. Following each time step, the distance between individuals was computed. If the distance between two pen mates was below a preset sensing range $r$, then the probability of a social interaction taking place was calculated from Eq. (5) and a binary record was sampled (1 means they interact, 0 means that they meet but do not interact).

For each interaction during the simulation process, the performer’s ID, recipient’s ID, performer’s position, recipient’s position, and pen ID were recorded. For each pair of pen mates, we also recorded the total number of encounters ${N}_{ij}$, which represents the number of potential interactions between $i$ and $j$, i.e. the number of time steps that the distance between individuals $i$ and $j$ was below the sensing range $r$.

### Determining realistic genetic input values

To obtain realistic results with respect to the accuracy of EBV and the sample size required to estimate genetic parameters, we mimicked real populations as good as possible, particularly with respect to the heritability of the trait. Observed-scale heritabilities for binomial traits related to animal behaviour are typically within the range of 0.05 to 0.20 [11–13]. To find realistic input values for our simulations, we simulated a test dataset where individuals performed on average 25 interactions (corresponding to ~ 2500 encounters per individual, each with a 1% probability of interaction), then estimated the observed-scale heritability with an ordinary linear mixed model, and next tuned the input value for the underlying genetic variance until the observed-scale heritability was 0.05, 0.1, or 0.2. These input values were then used for the actual simulations. In the simulation of the test dataset, we used a genetic correlation of zero between the traits $\alpha$ and $\beta$, noting that this only applied to the test set used to find realistic input values. In the simulation for genetic analysis we used three levels for the genetic correlation; see below in the section entitled “Implementation” section.

The following linear mixed model was used to estimate the observed-scale heritability from the test dataset:

10 $${n}_{i}=\mu +{A}_{i}+{\varepsilon }_{i},$$

where the response variable ${n}_{i}$ is the total number of interactions that $i$ performed, $\mu$ is the population mean, ${A}_{i}$ is the animal’s random genetic effect, and ${\varepsilon }_{i}$ is the residual. ASReml 4.1 [32] was used to fit the model and estimate the genetic (${\widehat{\sigma }}_{A}^{2}$) and residual (${\widehat{\sigma }}_{\varepsilon }^{2}$) variances. Observed-scale heritability was then estimated as:

11 $${\widehat{h}}_{o}^{2}=\frac{{\widehat{\sigma }}_{A}^{2}}{{\widehat{\sigma }}_{A}^{2}+{\widehat{\sigma }}_{\varepsilon }^{2}}.$$

Because we used equal genetic and permanent environmental variances for both traits (${\sigma }_{A,\alpha }^{2} ={ \sigma }_{A,\beta }^{2}={\sigma }_{Ep,\alpha }^{2}={\sigma }_{Ep,\beta }^{2}$; see section “Implementation” section below), the observed-scale heritability is also the same for both traits. Therefore, we identified the input values to obtain the desired observed-scale heritability for trait $\alpha$ and then used the same values for trait $\beta$. The resulting genetic parameters used for the final simulations are in Table 1.

**Table 1:** Simulated genetic variances (${{{\sigma}}}_{{{A}}}^{2}$)^a^ required to obtain a certain observed-scale heritability (${{{h}}}_{{{o}}}^{2}$)^b^

${h}_{o}^{2}$	${h}_{u}^{2}$	${\sigma }_{A}^{2}$	$\overline{p }({A}_{\alpha }={\mu }_{\alpha }-2{\sigma }_{{A}_{\alpha }})^\text{c}$	$\overline{p }({A}_{\alpha }={\mu }_{\alpha }+2{\sigma }_{{A}_{\alpha }})^\text{c}$	Accuracy LMM^d^
0.05	0.0036	0.012	0.008	0.012	0.173
0.1	0.0111	0.038	0.006	0.015	0.195
0.2	0.0429	0.170	0.004	0.022	0.241

^a^Variances were the same for genetic effect and permanent environmental effect, as well as for both traits, so these input values are for all four variances

^b^ ${h}_{o}^{2}$ is the observed-scale heritability of the mean of 2500 binary observations with on average 25 successes, based on a linear mixed model

^c^Interaction probability of the top and bottom ranking individuals for the performer effect. These values are calculated assuming that an individual whose value for trait $\alpha$ is ${P}_{\alpha }={\mu }_{\alpha }\pm 2{\sigma }_{{A}_{\alpha }}$ interacts with an average pen mate (${P}_{\beta }={\mu }_{\beta }$). The interaction probability for such a combination is $p=$ logistic (${\mu }_{\alpha }\pm 2{\sigma }_{{A}_{\alpha }}+{\mu }_{\beta }$). The mean interaction probability was 1% (${\mu }_{\alpha }={\mu }_{\beta }=1/2logi{stic}^{-1}\left(0.01\right)$)

^d^Accuracy of the EBV for the performer effect based on the simple LMM, computed as the correlation between true and estimated breeding values

### Genetic analysis

Next, we investigated sample sizes required for accurate estimation of genetic parameters and associated bias and accuracy of EBV. To estimate genetic parameters and BV for traits $\alpha$ and $\beta$, the following generalized linear mixed model (GLMM) with a logit link-function and a binomial distribution was fitted in ASReml 4.1 [32], using the data of the offspring generation:

12 $$logit\left({p}_{ij}\right)=\mu +{Pen}_{k}+{A}_{\alpha ,i}+{A}_{\beta ,j}+{Ep}_{\alpha ,i}+{Ep}_{\beta ,j},$$

where ${p}_{ij}$ is the probability that animal $i$ performed a social interaction towards $j$ given their distance is below the sensing range $r$ (i.e. they encounter). ${Pen}_{k}$ is a fixed pen effect, ${A}_{\alpha ,i}$ is the random BV of individual $i$ for performer effect, ${A}_{\beta ,j}$ is the random BV of individual $j$ for recipient effect, and ${Ep}_{\alpha ,i}$ and ${Ep}_{\beta ,j}$ are the corresponding independently distributed random permanent environmental effects. Co-variances of breeding values among individuals were based on the pedigree relationship matrix $\mathbf{A}$:

13 $$var\left[\begin{array}{c}{\mathbf{a}}_{\alpha }\\ {\mathbf{a}}_{\beta }\end{array}\right]=\left(\begin{array}{cc}{\sigma }_{{A}_{\alpha }}^{2}& {r}_{A}{\sigma }_{{A}_{\alpha }}{\sigma }_{{A}_{\beta }}\\ {r}_{A}{\sigma }_{{A}_{\alpha }}{\sigma }_{{A}_{\beta }}& {\sigma }_{{A}_{\beta }}^{2}\end{array}\right)\otimes {\mathbf{A}}^{-1},$$

where $\otimes$ denotes the Kronecker product. In these analyses, we only included records for pairs of individuals that met at least once. Hence, pairs of individuals that did not meet were omitted. For each replicate, we estimated both the genetic parameters and the BV. Hence, the quality of EBV will also reflect the quality of the estimated genetic parameters. The quality of the EBV was evaluated by their accuracy, the correlation between the true BV (TBV) and EBV, and bias, the regression of the BV on EBV.

Two traits were defined in this study, i.e. the performer and recipient effects. For each individual, the sum of the BV for these two traits was defined as the total BV [22], i.e.:

14 $$total\; BV=B{V}_{\alpha }+B{V}_{\beta }, total\; EBV=EB{V}_{\alpha }+EB{V}_{\beta }.$$

We focused on the total BV because it reflects response to selection, which is the ultimate purpose of animal breeding. In other words, the per-generation change in mean total BV is equal to the response to selection in the underlying trait value, i.e., in ${\overline{P} }_{\alpha }+{\overline{P} }_{\beta }$. Note that individuals with a lower total BV will show less harmful social interactions, such that lower values of the total BV are preferable. In the remainder of this paper, reported accuracies and biases refer to the total EBV, unless explicitly stated otherwise, i.e.:

15 $$Accuracy=cor\left(total\; BV, total\; EBV\right),$$

16 $$Bias=reg\left(total\; BV, total\; EBV\right).$$

### Implementation

#### The default scenario

We considered a relatively small population, as automated detection of individual social behaviours with sensors and AI is still under development. In the default scenario, 2000 offspring were generated from 100 sires and 400 dams, where each sire was mated to four dams and each dam produced five offspring. Then, the 2000 offspring were randomly divided into 20 groups of 100 individuals. Hence, there were 9900 ordered pairs of individuals in each pen (i.e., 100 × (100–1) i.e. when distinguishing $ij$ from $ji$. The mean trait value on the underlying scale for each trait (${\mu }_{\alpha }$ and ${\mu }_{\beta }$) was set to -2.3 to result in a median interaction probability of 1% given that individuals encountered each other (such that ${\mu }_{\alpha }+{\mu }_{\beta }=logit(0.01)$). The simulation was run until at least 10,000 interaction events took place in each pen. Given the average interaction probability of 1%, this implied around 1 million encounters per pen (a value realistic for actual populations; see “Discussion” section). By the end of the simulation, the total number of encounters and the total number of interactions were determined for all 9900 × 20 pairs of individuals. The simulations were replicated 20 times, and results are based on the averages of these 20 replicates.

As mentioned above, the variance of the BV and permanent environment effects were assumed equal for both traits (Table 1). Three levels for the genetic correlation between trait $\alpha$ and trait $\beta$ were simulated, -0.5, 0, and 0.5. When the two traits are positively correlated, individuals that perform more interaction behaviours also tend to receive more interactions from others, and vice versa.

Pen size followed from a fixed density of one animal per m^2^. Thus, pen size was 100 m^2^ in the default scenario (10 m × 10 m). For each pen, 20 feeders were uniformly located on two opposite sides of the pen (i.e. 40 in total). In each time step, an animal moved 1 m if it was motivated for walking, either in a random direction or towards a feeder when motivated for eating.

To obtain the behavioural pattern described in Fig. 1a, the threshold $T$ was set at 100 and the matrix of changes of motivation per time step was set at:

18 $${\Delta }_{t,t+1}=\left(\begin{array}{ccc}-1& 0.025& 0.025\\ 0& -10& 10\\ 0& 10& -10\end{array}\right).$$

#### Alternative scenarios

Compared to the default scenario, three parameters were varied, one at a time: (a) the total number of individuals, (b) the number of observed interactions per pen, and (c) group size.

- To evaluate the impact of the total number of individuals, we varied the number of pens included in the statistical analysis from 1 to 20 (where 20 was the default). Thus, the number of animals ranged from 100 to 2000. To accomplish this, we did not change the simulations but assumed data were available from a range for numbers of pens. Because families were distributed randomly across pens, variation in the number of pens included in the analysis resulted in variation in the amount of sib information included in breeding value estimation. In the default situation with 2000 individuals, an animal has own performance and records of four full sibs and 15 half-sibs. When the number of individuals was reduced to 100, an average individual had own performance and records on 0.2 full-sibs and 0.75 half-sibs.
- To evaluate the impact of the number of observed interactions per pen, we extended the simulated time until at least 100k interactions were obtained for each pen. The GLMM was then fitted by including the first 5k, 10k (default), 20k, 50k, or 100k interactions.
- To evaluate the impact of group size, we randomly divided the 2000 individuals into 10, 20 (default), 50, or 100 groups. The corresponding group sizes were 200, 100 (default), 40 and 20 individuals. The average number of interaction events per individual was set at 100 for each group size. Hence, simulations were continued until the number of interactions recorded in each pen was at least 100 × group size. With ${n}_{pens}$ pens of $n$ individuals, yielding a total of $N={n}_{pens} n$ individuals, the total number of (ordered) pairs that can potentially interact is equal to $N(n-1)$. Hence, for a given $N$, the number of pairs decreased as pen size decreased, which may affect the accuracy of EBV. Because density was fixed, larger groups were simulated using larger pens and the total area summed over all pens was always 2000 m^2^, irrespective of the number of groups.

## Results

### Default scenarios

Table 1 shows the genetic variances (${\sigma }_{A}^{2}$) that correspond to observed-scale heritabilities of total number of interactions performed (${h}_{o}^{2}$) of 0.02, 0.05, and 0.1, and the corresponding heritabilities on the underlying scale. Note that the underlying-scale heritability is defined here for a single realization of the latent variable, while the observed-scale heritability is estimated from the sum of ~ 2500 Bernoulli trials, resulting in an average of ~ 25 interactions per individual (see “Methods” section). Thus, the random sampling process has much less impact on the observed-scale heritability, which explains why ${h}_{o}^{2}$ is much greater than ${h}_{u}^{2}$. To interpret the magnitude of the genetic variance on the underlying scale, we also show the corresponding expectation of interaction probabilities for assumed individuals whose BV were $2{\sigma }_{A}$ above or below average (Table 1). The interaction probabilities for the top and bottom ranking individuals differed by a factor of 1.5 for ${h}_{o}^{2}$ = 0.05, 2.5 for ${h}_{o}^{2}$ = 0.10, and 5.5 for ${h}_{o}^{2}$ = 0.20, assuming that these top/bottom animals interact with random pen mates. Hence, in spite of the low observed-scale heritability, the genetic differences between individuals in their tendency to engage in social interactions were substantial. Accuracies of the EBV for the performer effect predicted by the ordinary LMM were relatively small (Table 1). Because simulated variance components were equal for the performer and recipient effects, results for the recipient effects (not shown) were very similar to those shown in Table 1.

The results also show that estimated genetic parameters did not differ from their true values (Table 2 for ${r}_{A}$ = 0, and see Additional file 2: Table S1 for ${r}_{A}=-0.5,$ and for ${r}_{A}=0.5$). In this analysis, we assumed that ${\sigma }_{{A}_{\alpha }}^{2}$, ${\sigma }_{{A}_{\beta }}^{2}$, ${\sigma }_{{Ep}_{\alpha }}^{2}$, and ${\sigma }_{{Ep}_{\beta }}^{2}$ have the same true value in order to simplify the simulation, so similar estimates were expected.

**Table 2:** Estimated and true genetic parameters based on the GLMM for the default scenario with ${{{r}}}_{{{A}}}=0$

${r}_{A}=0$	${h}_{o}^{2}$					
	0.05		0.1		0.2	
	Estimated	True^a^	Estimated	True^a^	Estimated	True^a^
${\sigma }_{{A}_{\alpha }}^{2}$	0.0121	0.012	0.0396	0.038	0.1719	0.170
${\sigma }_{{A}_{\beta }}^{2}$	0.0138	0.012	0.0375	0.038	0.1706	0.170
${\sigma }_{{Ep}_{\alpha }}^{2}$	0.0117	0.012	0.0358	0.038	0.1649	0.170
${\sigma }_{{Ep}_{\beta }}^{2}$	0.0113	0.012	0.0361	0.038	0.1655	0.170
${r}_{A}{\sigma }_{{A}_{\alpha }}{\sigma }_{{A}_{\beta }}$	0.0009	0	0.0012	0	– 0.0004	0

Estimates were averages of 25 replicates.

^a^The true values of variances were the simulated input values (Table 1).

${r}_{A}{\sigma }_{{A}_{\alpha }}{\sigma }_{{A}_{\beta }}$ is the genetic covariance between the two traits.

For the default scenario, the accuracies of the EBV for the two latent traits were slightly higher than the accuracy of the total EBV (Table 3).

**Table 3:** Comparison of accuracies for total versus latent trait EBV for the default scenario^a^

${r}_{A}=0$	${h}_{o}^{2}$		
	0.05	0.1	0.2
${r}_{IH,total}$	0.471	0.595	0.713
${r}_{IH,\alpha }$	0.532	0.624	0.727
${r}_{IH,\beta }$	0.516	0.638	0.718

^a^Accuracies refer to the default scenario, where the number of phenotyped individuals is 2000, the average number of records per individual is 10, and the group size is 100

Table 4 shows the accuracy and bias of the total EBV from the GLMM for the default scenario for nine combinations of three observed-scale heritabilities (${h}_{o}^{2} = 0.05, 0.1, \mathrm{and}\,0.2$) and three genetic correlations between performer and recipient effects (${r}_{A} = -0.5, 0,\mathrm{ and }\,0.5$). Accuracies were moderate and increased with heritability. For example, for ${r}_{A}$ = 0, the accuracy was 0.47, 0.60, and 0.71 for ${h}_{o}^{2}$ of 0.05, 0.1, and 0.2, respectively. Breeding values were overestimated by 6 to 15% and bias was larger when heritability was lower.

**Table 4:** Accuracy and bias of EBV from the GLMM for the default scenario

${h}_{o}^{2}$		${r}_{A}$		
		− 0.5	0	0.5
0.05	Accuracy	0.455 (± 0.009)	0.471 (± 0.009)	0.448 (± 0.008)
	Bias	0.875 (± 0.023)	0.847 (± 0.027)	0.881 (± 0.016)
0.1	Accuracy	0.571 (± 0.008)	0.595 (± 0.011)	0.563 (± 0.015)
	Bias	0.917 (± 0.019)	0.911 (± 0.022)	0.896 (± 0.018)
0.2	Accuracy	0.668 (± 0.012)	0.713 (± 0.017)	0.659 (± 0.010)
	Bias	0.936 (± 0.030)	0.925 (± 0.019)	0.928 (± 0.021)

Standard errors are in brackets. For the bias, regression coefficients of true total breeding values on estimated total BV are shown, so that values smaller than 1 indicate overestimation of breeding values. ${h}_{o}^{2}$ is the observed-scale heritability of the mean of 2500 binary observations with on average 25 successes, based on a linear mixed model

### Number of phenotyped individuals

**Fig. 2:**
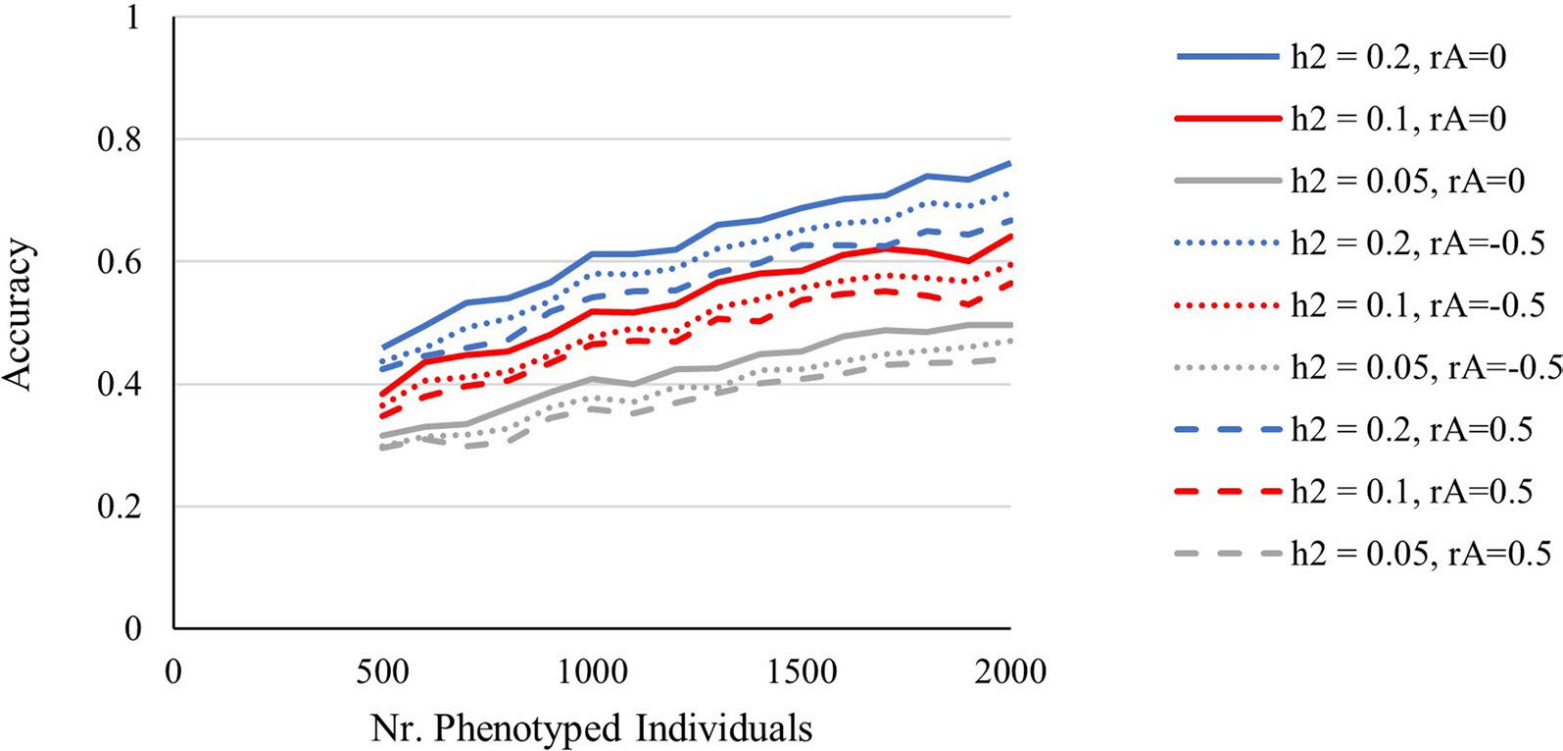
Accuracy of EBV as a function of the number of phenotyped individuals. For different observed-scale heritabilities and genetic correlations between performer and recipient effect. The number of individuals was varied by varying the number of groups included in the analysis. Statistical analysis did not converge with fewer than 500 individuals

Results for the effect of the number of phenotyped individuals on accuracy of EBV are shown in Fig. 2. With less than 500 individuals, the size of the dataset was too small to fit the GLMM for some replicates using ASReml and we did not test whether this issue could be resolved with other software. Note that for each replicate, we not only estimated BV but also the variance components. When the phenotypes of 500 individuals were included, ASReml successfully fitted the GLMM for all 20 replicates and accuracies were 0.30, 0.36, and 0.44 for ${h}_{o}^{2}$ of 0.05, 0.1, and 0.2, respectively (Fig. 2). As the number of phenotyped individuals increased to 2000, the accuracy of EBV increased to 0.47, 0.59, and 0.71 for ${h}_{o}^{2}$ of 0.05, 0.1, and 0.2, respectively (Fig. 2). Hence, with 2000 individuals, accuracies of ~ 0.5 or more were obtained, suggesting that 50% or more of the maximum possible response to selection can be obtained. Thus, results in Fig. 2 show that promising accuracies can be obtained with relatively small datasets, although genetic parameters needed to be also estimated for each replicate.

### Number of observations

**Fig. 3:**
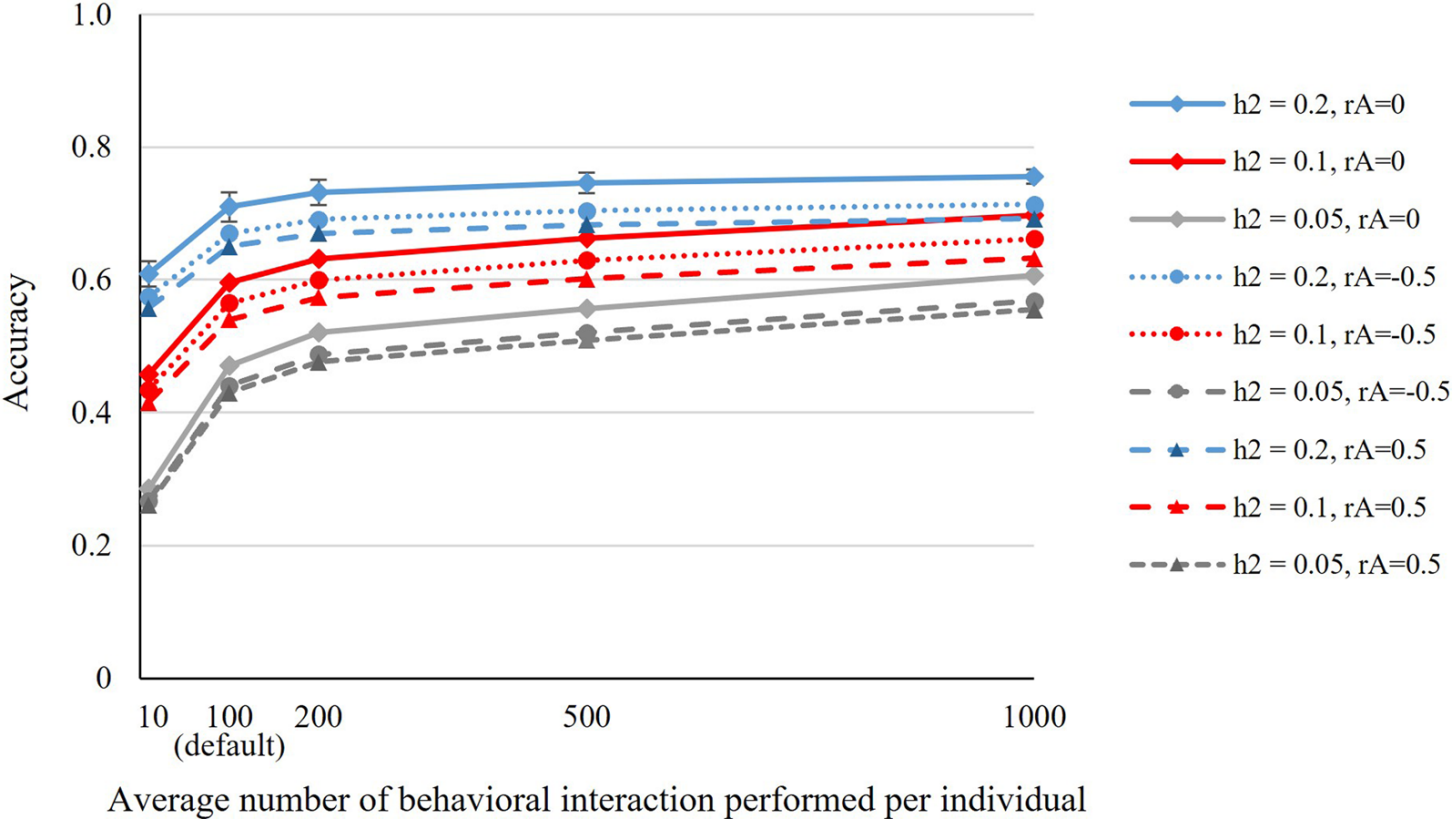
The accuracy of EBV as a function of the number of records per individual. For a fixed number of pens (20) and a fixed number of individuals per pen (100). In total, 100 thousand encounters were simulated, and the first 5, 10, 20, 50, 100 thousand records were included in the statistical analysis. Error bars (± 1 SE) are shown for the top line only; SE for the other lines were similar

Results show that the accuracy increased with the increasing number of records, as expected (Fig. 3). However, as the amount of data increased, the accuracy increased at a diminishing rate, and the benefit of additional data decreased, particularly with higher heritabilities. Note that accuracy does not asymptote to a value of 1 because of the permanent environmental components of the individual phenotype, which had the same variance as the genetic effects. A derivation of the theoretical upper bound of the accuracy when including an infinite amount of data is in Discussion and (see Additional file 3: Text S2).

### Group size

**Fig. 4:**
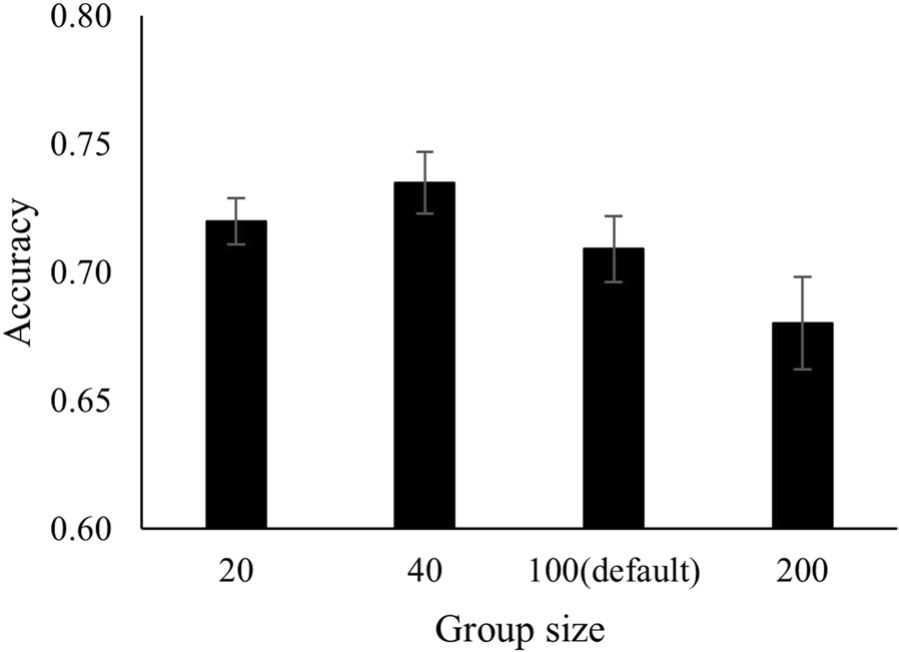
Effect of group size on the accuracy of EBV. For a total of 2000 individuals and a pen size of 20, 40, 100, or 200 individuals. Error bars indicate ± 1 SE. In panels a and b, ${h}_{o}^{2}$ is the observed-scale heritability of the mean of 2500 binary observations with on average 25 successes, based on a linear mixed model. In all panels, accuracies are averages of results for the performer and the recipient trait

Group size had only a minor impact on the accuracy of EBV (Fig. 4). In the four scenarios with different group sizes, accuracy ranged from 0.68 (group size of 200) to 0.73 (group size of 40). Thus, a larger group size led to a slight decrease in accuracy. However, the difference in accuracy was statistically significant only between group sizes 40 and 200 (*P* < 0.05). The average number of social interactions per individual was identical for the four group sizes. However, individuals interact with more pen mates in scenarios with a larger group size. Therefore, with a fixed total number of records, the average number of records per pair of individuals is smaller in larger groups. This can become problematic when the total number of records is small and a lot of zeros show up in the data, which may lead to poor estimation of probabilities and BV. In these cases, one could record more interaction events or use statistical techniques to overcome this issue.

## Discussion

### Performence of the analysis model

We investigated prospects for genetic improvement of social traits recorded in large livestock populations with hundreds of animals. For this purpose, we developed an individual-based simulation method to generate data on social interactions in a population with individual genetic and permanent environmental effects, and with spatial movement of individuals over time. We also presented a GLMM to estimate genetic parameters and BV for performer and recipient effects from the simulated data. We chose input values to ensure that estimated observed-scale heritability agrees with typical values of the heritability for social behaviour traits found in the literature. Thus, we believe that our simulations reflect the magnitude of genetic effects in actual livestock populations. These low observed-scale heritabilities corresponded to relatively large genetic differences between individuals in the tendency to express a behaviour.

The GLMM successfully estimated genetic parameters that were not significantly different from the true values. Results also showed a considerable accuracy of total EBV. With the half-sib family structure used in this study and the presence of permanent environmental effects, the maximum possible accuracy is 0.77 (see Additional file 3: Text S2). The realized accuracy of 0.71 with ${h}_{o}^{2}=0.2$ and ~ 100 interactions per animal is already very close to this upper bound. For the other scenarios, more records were required to approach the upper bound. With a very large number of sibs, the theoretical upper bound of accuracy would be 0.82 (see Additional file 3: Text S2), which is still only a little larger than the 0.71 value we found for ${h}_{o}^{2}=0.2$.

Significant biases of EBV were observed in all scenarios. One reason for these biases could be inaccurate estimation of genetic parameters; when the true values of the genetic parameters were used in the GLMM instead of the estimates, the bias of EBV became non-significant in four out of nine scenarios.

The amount of data that we used, i.e. a total of 10,000 interactions per pen of 100 individuals, may seem large and unrealistic. However, literature on the frequency of behavioural interactions in livestock suggests that this amount of data corresponds to several days or weeks of observation, rather than months or years. For laying hens, for example, Blokhuis et al*.* [33] found a frequency of feather pecking ranging from 28 to 74 pecks per hour per individual. For swine, Brunberg et al*.* [34] found that a single fattening pig performed 0 to 80 tail bites per hour and received 0 to 30 tail bites per hour. Our default scenario included on average 100 interactions per individual. Even with only one interaction per individual per hour, our data would correspond to no more than 100 h of recording. Even when animals are active only during the day, this amount of data can be collected in a few weeks.

The GLMM yielded higher accuracies of EBV than the ordinary LMM but we believe this is due to an unequal amount of data that was used to fit these two models. The LMM was only used to tune the input values to obtain realistic observed-scale heritabilities. Therefore, for the test dataset, we used an amount of data similar to that in previous studies, i.e., 25 interactions per animal on average. With the GLMM, in contrast, our aim was to investigate the accuracy of EBV for datasets obtained with computer vision and AI and, as a result, we used 100 interactions per animal. Thus, results of the GLMM and LMM cannot be compared. We used the GLMM to estimate BV because it is theoretically more appropriate for count data than the LMM and because it can account for variation in the potential number of interactions between pairs of individuals (i.e., in the number of encounters). when the amount of data is smaller, random variation in the number of encounters among pairs of individuals will be larger and the number of interactions per pair will deviate more from a normal distribution. Thus, when the amount of data is limited, we expect GLMM to be more suitable for dealing with the resulting nonlinear relationship between the frequency of social interactions and underlying BV. When the amount of data is large enough such that the interaction frequencies are close to a normal distribution and all pairs of individuals have ample interaction, we expect little difference between the accuracy of EBV for performer and recipient effects obtained from LMM and the GLMM we used here. Some additional results shown in Table 5 for a single scenario are in line with this expectation, suggesting that the GLMM is superior when the amount of data is limited.

**Table 5:** Comparison of the accuracy of EBV based on LMM and GLMM with different amounts of data^a^

Model	Number of behavioural interactions per animal		
	10	100	1000
LMM	0.196	0.340	0.633
GLMM	0.609	0.710	0.756

^a^ $$n=2000$$, $${h}_{o}^{2}=0.2$$ and $${r}_{A}$$ = 0

### Impact of the investigated parameters

We investigated the effects of the number of phenotyped individuals on the accuracy of EBV. For this comparison, we assumed a situation in which a large population is housed in several pens (with random grouping of animals over pens) and where the video monitoring system is installed by pen. We found that a larger number of phenotyped individuals resulted in a higher accuracy (Fig. 2). Thus, equipping more pens with the video system resulted in higher accuracy of EBV. This increase of the accuracy of EBV with population size can be partly explained by the associated increase in the number of relatives in the data. When more individuals are phenotyped, more sib information will be included. When all 20 pens were phenotyped, each individual had records for four full sibs and 15 half sibs. However, when only half of the pens was phenotyped, sib information was also halved, which led to lower accuracies (from 0.71 to 0.61). More precise estimation of genetic parameters may also have contributed to the higher accuracy of EBV with larger populations [35].

In terms of the number of records, we found that ~ 100 interactions per animal yielded a fairly high accuracy of EBV (0.71) and increasing the number of interactions tenfold increased accuracy by only 7% (0.78). Given that automated phenotyping is still relatively complicated and costly, we suggest to increase the number of phenotyped individuals rather than increase the number of interactions recorded per individual (if ~ 100 interactions per individual are available). One way to achieve this is to equip a few pens with a system for automated behaviour detection, and to replace the individuals in those pens, for example, every few weeks. Nevertheless, a key challenge remains to collect a sufficiently large amount of annotated data to train AI algorithms for automated behaviour detection.

We found a minor effect of group size on the accuracy of EBV. Although there was a statistically significant difference for one of the comparisons (n = 40 vs. n = 200), differences in accuracy were still minor. In larger groups, animals can interact with more group mates but the number of interactions for specific pairs will be smaller (as we assumed a fixed total number of interactions). Apparently, these two opposing effects largely compensate each other. Given the minor impact of group size on accuracy, we suggest that group size should mainly be determined by practical considerations.

### Impact of other parameters

A variety of factors play a role in social interactions. However, due to the lack of quantitative data on these factors, it is difficult to incorporate them into the simulation at present. Therefore, we made a number of assumptions in this study. First, we assumed that animal movement is completely random and we did not simulate genetic differences in movement between animals. However, genetic differences in movement between individuals probably exist and more active animals will have more opportunity to interact with pen mates. Factors such as walking speed and resting time may also show large variation between individuals [36, 37], which should also be taken into account in future research. Particularly if individual variation in movement has a genetic basis, it may be relevant to include such variation in the breeding value estimation, both to optimize response to selection in the behaviours of interest, and to prevent undesirable correlated responses.

Second, animals typically develop a hierarchy, which affects their role in social interactions, but we did not include this in this study. Animals may carefully assess their opponent and take into account what happened in previous interactions before deciding how to interact [38]. Moreover, research shows that the number of interactions is typically the largest when animals are mixed and the hierarchy is not yet formed [39, 40]. During this time, animals explore the pen and interact with the pen mates to adapt to the new environment and to establish a dominance hierarchy. Less interaction will be performed after establishment of hierarchy and animals with higher hierarchy are more frequently a performer than a receiver. In future studies, establishment of hierarchy should be included by, e.g., using a feedback loop where the animal's hierarchy will decrease if the animal is frequently at a disadvantage in interactions. The hierarchy could also be analyzed by, for example, social network analysis (SNA), which has recently been applied to relatively large animal populations [26]. In social network models, individuals are nodes and their interactions are edges connecting two nodes. SNA is able to provide novel centrality traits that describe an individual’s role in group interactions and, using these, one can take the impact of hierarchy further into account.

Third, we assumed that the (expected) frequency of social interactions is constant for a pair of animals, while it has been shown that social behaviours can spread across the group like an epidemic [41, 42]. This phenomenon is called social transmission and suggests that social behaviours are not independent among individuals but may show positive feedbacks. In laying hens, for example, severe feather pecking can cause naked areas and wounds on the recipient [43], which are attractive to pen mates and may cause increased pecking on the victim. This factor is similar to hierarchy, as both are related to the dynamics of individual behaviour over time and both cause individuals that are more involved in interactions to tend to become engaged in even more interactions in the future. We think that SNA can also be used to analyze such social transmission. Assuming hierarchy is established within the group and is fixed, the phenomenon that one may observe as a result of social transmission is a gradual increase of edges connecting nodes because animals copy the behaviour of others.

All above-mentioned factors have been proven to exist but there is still a lack of quantitative studies, so they were not included in the present study. However, rapid developments in AI and computer vision techniques promise to deliver quantitative information on these factors in the near future [26, 28]. In the future, if we are able to take these factors into account, a relevant issue that needs to be addressed is that hierarchy and social transmission will make the interaction records dependent because both describe the phenomenon that individuals act according to previous experience. Dependent records lead to overdispersion of binomial interaction records, which is problematic, for example, because it leads to false statistical significance. Including appropriate random effects, such as a temporal environmental term, may help to account for overdispersion. To accommodate this, we recommend the data collection period to be extended and use intermittent records. For example, if 14 days of data are needed, instead of collecting the data continuously for two weeks, it might be preferable to collect data for two days per week for a period of seven weeks since the animals were mixed. With this strategy, it may also be possible to analyze the establishment of hierarchy and the spread of behaviour, and include time-dependent temporal environmental effects for each combination of individuals.

Given the accuracy of EBV that we found and given the fact that relevant data will become available in the near future, genetic improvement appears to be a promising strategy to improve social behaviours in livestock. However, there is still lack of knowledge on the genetic basis of directional social behaviours and on the relationship between behavioural traits and other traits. Animals that show less harmful social behaviour may be less fearful or less stressed animals but could, for example, also be inactive or dim-sighted. Although the first results on selection for indirect genetic effects in pigs and laying hens suggest the opposite [44], it is necessary to pay attention to changes in other traits when selecting for behavioural traits.

## Conclusions

We investigated prospects for genetic improvement of social traits. For this purpose, we developed methods to simulate social behaviours and to analyze the resulting data. Results showed unbiased genetic parameter estimates and promising accuracies of EBV of social tendency, even with only ~ 100 interactions per individual, which corresponds to a few weeks of recording. The analysis model is applicable to large-scale longitudinal data on behavioural interactions between animals kept in large groups. Given the facts that (1) phenotype data will become available in the near future and (2) genetic analysis of these phenotype data is feasible, we conclude that animal breeding can be a promising strategy to improve social behaviours in the future.

### Supplementary Information

**Additional file 1: Text S1.** Determination of rate of motivation for eat, walk and rest behaviour. The file explains how matrix ${\Delta }_{\mathbf{t},\mathbf{t}+1}$ was determined to fulfill a target realistic behaviour pattern similar. **Figure S1.** Simulated behavioural pattern of the individuals.

**Additional file 2: Table S1.** Estimation of genetic parameters, accuracy and bias of EBV from GLMM for all combinations of ${h}_{o}^{2}$ and ${r}_{A}$. The table contains complete estimated genetic parameters, accuracy and bias for all of the set-ups.

**Additional file 3: Text S2.** Estimation of the upper bound of accuracy in the GLMM. The file shows the calculation of the upper bound of the accuracy one could get with ${\sigma }_{A}^{2}={\sigma }_{Ep}^{2}$.

## Data Availability

If the manuscript is accepted we will provide the scripts in a repository upon request. No actual data were used for this study.
