# Supplementary material for: Towards genetic improvement of social behaviours in livestock using large-scale sensor data: data simulation and genetic analysis
Source: Genet Sel Evol. 2023 Sep 28;55:67. doi: 10.1186/s12711-023-00840-z (PMC10537099; doi:10.1186/s12711-023-00840-z)
Supplement: Supplementary file 1 — Additional file 1: Text S1. Determination of rate of motivation for eat, walk and rest behaviour. The file explains how matrix ${\Delta }_{\mathbf{t},\mathbf{t}+1}$Δt,t+1 was determined to fulfill a target realistic behaviour pattern similar. Figure S1. Simulated behavioural pattern of the individuals. [file 12711_2023_840_MOESM1_ESM.docx]

**Additional file 1**

**Determination of rate of motivation for eat, walk and rest behavior**

In this work, three motor behaviors were defined and each individual has three status: eat(E), walk(W) and rest(R). Three motivations were therefore defined: $M_{E}, M_{W},M_{R}$. Together they make up the motivation vector:

$$\boldsymbol{m}_{i,t}=\left( \begin{matrix} M_{Ei,t} \\ M_{Wi,t} \\ M_{Ri,t} \end{matrix} \right)$$

The change of motivation per step is given by the following matrix:

$$\boldsymbol{\Delta}_{t,t+1}=\left( \begin{matrix} \begin{matrix} \Delta_{E\leftarrow E} & \Delta_{E\leftarrow W} & \Delta_{E\leftarrow R} \end{matrix} \\ \begin{matrix} \Delta_{W\leftarrow E} & \Delta_{W\leftarrow W} & \Delta_{W\leftarrow R} \end{matrix} \\ \begin{matrix} \Delta_{R\leftarrow E} & \Delta_{R\leftarrow W} & \Delta_{R\leftarrow R} \end{matrix} \end{matrix} \right)_{t,t+1}$$

$\Delta_{E\leftarrow E}$is the change of eating motivation when animal is eating.

$\Delta_{W\leftarrow E}$is the change of walking motivation when animal is eating.

After one step, motivation vector is updated accordingly:

$$\boldsymbol{m}_{i,t+1}=\boldsymbol{m}_{i,t}+\left( \begin{matrix} \Delta_{E\leftarrow B^{'}} \\ \Delta_{W\leftarrow B^{'}} \\ \Delta_{R\leftarrow B^{'}} \end{matrix} \right), B^{'}=E,W,R.$$

Then the program would check that was any motivations above threshold T (T=100).We should design $\Delta_{t,t+1}$ properly to fulfill an assumed realistic behavior pattern similar. Animal will perform a behavior when the motivation reach threshold ($M_{Behavior}>T$), and will stop when the motivation is below zero ($M_{Behavior}<0$). Change of motivation for this performing behavior are diagonal elements in$\boldsymbol{\Delta}_{t,t+1}$.

Let ${Duration}_{E}, {Duration}_{W},{Duration}_{R}$ be the average duration time of single continuous meal walk and rest. Then the relationship between duration time and $\boldsymbol{\Delta}_{t,t+1}$ is:

${Duration}_{E}= -\frac{T}{\Delta_{E\leftarrow E}}$ (1)

${Duration}_{W}= -\frac{T}{\Delta_{W\leftarrow W}}$ (2)

${Duration}_{R}= -\frac{T}{\Delta_{R\leftarrow R}}$ (3)

Now we have three equations and the diagonal elements can be determined.

In the simulation, after a couple of steps (each behavior have occurred at least twice), the animal’s behavior pattern will become relatively fixed and it will repeat the behavior pattern. In reality, eating behavior happens less than walk and rest. Between two eating behavior, the animal would switch between walk and rest for several times .


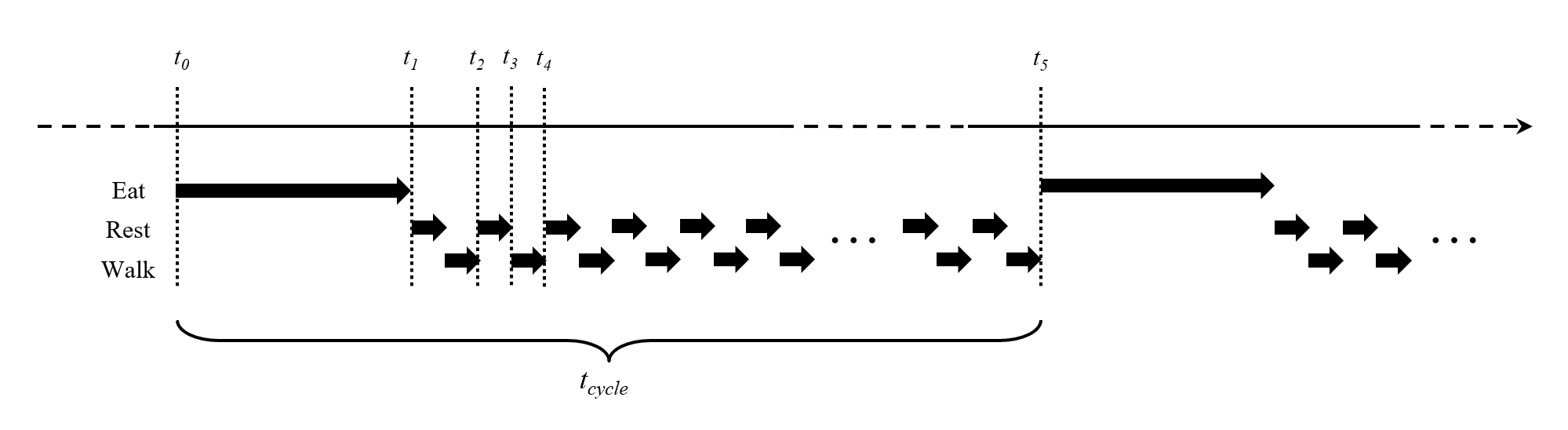


**Figure S1.** **Simulated behavioral pattern of the individuals.**

See figure above. A eat behavior start at t_0_ and end at t_1_, followed by a couple of walks and rests. At t_5_, eating behavior start again. The process from t_0_ to t_5_ is a cycle.

Let $t_{cycle}=t_{5}-t_{0}$ be the average time between meals.

Let $p_{E},p_{W},p_{R}$ be the overall proportion of time that animal spend on each behavior.

At t_0_ and t_5_, the individual status are identical, therefore motivation change is zero.

$$\mathbf{m}_{\mathbf{t}_{\mathbf{5}}}-\mathbf{m}_{\mathbf{t}_{\mathbf{0}}}= \boldsymbol{\Delta}_{t,t+1}\boldsymbol{\times}\left( \begin{matrix} t_{cycle}*p_{E} \\ t_{cycle}*p_{W} \\ t_{cycle}*p_{R} \end{matrix} \right)\mathbf{=0}$$

Or

$$\begin{aligned} \left( \begin{matrix} \begin{matrix} \Delta_{E\leftarrow E} & \Delta_{E\leftarrow W} & \Delta_{E\leftarrow R} \end{matrix} \\ \begin{matrix} \Delta_{W\leftarrow E} & \Delta_{W\leftarrow W} & \Delta_{W\leftarrow R} \end{matrix} \\ \begin{matrix} \Delta_{R\leftarrow E} & \Delta_{R\leftarrow W} & \Delta_{R\leftarrow R} \end{matrix} \end{matrix} \right)\boldsymbol{\times}\left( \begin{matrix} p_{E} \\ p_{W} \\ p_{R} \end{matrix} \right)\mathbf{=0}\#\left( 4 \right) \end{aligned}$$

For rest behavior:

At t_2_, $M_{R,t_{2}}$=0.

At t_3_, $M_{R,t_{3}} = T$

From t_2_ to t_3_:

$$M_{R,t_{3}} - M_{R,t_{2}} = {Duration}_{W} * \Delta_{R\leftarrow W}$$

Note that: ${Duration}_{W}= -\frac{T}{\Delta_{W\leftarrow W}}$

Therefore:

$\Delta_{R\leftarrow W} = -\Delta_{W\leftarrow W}$ (5)

For walk behavior:

Similarly, from t_3_ to t_4_, motivation of walk increased from 0 to T, hence:

$\Delta_{W\leftarrow R} = -\Delta_{R\leftarrow R}$ (6)

For eat behavior:

From t_1_ to t_5_, motivation of eat increased from 0 to T:

$$M_{E,t_{5}} - M_{E,t_{1}} = T$$

Moreover,

$M_{E,t_{5}} - M_{E,t_{1}} = t_{cycle}p_{W} \Delta_{E\leftarrow W} + t_{cycle}p_{R} \Delta_{E\leftarrow R} =T$ (7)

With these equations, one can easily solve the $\boldsymbol{\Delta}_{t,t+1}$ matrix as a function of threshold ($T$), time between meals ($t_{cycle}$), duration of each behavior (${Duration}_{E}, {Duration}_{W},{Duration}_{R}$), and proportion of time spent on each behavior ($p_{E},p_{W},p_{R}$).

$${Duration}_{E}= -\frac{T}{\Delta_{E\leftarrow E}}$$

$${Duration}_{W}= -\frac{T}{\Delta_{W\leftarrow W}}$$

$${Duration}_{R}= -\frac{T}{\Delta_{R\leftarrow R}}$$

$$\left( \begin{matrix} \begin{matrix} \Delta_{E\leftarrow E} & \Delta_{E\leftarrow W} & \Delta_{E\leftarrow R} \end{matrix} \\ \begin{matrix} \Delta_{W\leftarrow E} & \Delta_{W\leftarrow W} & \Delta_{W\leftarrow R} \end{matrix} \\ \begin{matrix} \Delta_{R\leftarrow E} & \Delta_{R\leftarrow W} & \Delta_{R\leftarrow R} \end{matrix} \end{matrix} \right)\boldsymbol{\times}\left( \begin{matrix} p_{E} \\ p_{W} \\ p_{R} \end{matrix} \right)\mathbf{=0}$$

$$\Delta_{R\leftarrow W} = -\Delta_{W\leftarrow W}$$

$$\Delta_{W\leftarrow R} = -\Delta_{R\leftarrow R}$$

$$t_{cycle}p_{W} \Delta_{E\leftarrow W} + t_{cycle}p_{R} \Delta_{E\leftarrow R} =T$$
