# Supplementary material for: Towards genetic improvement of social behaviours in livestock using large-scale sensor data: data simulation and genetic analysis
Source: Genet Sel Evol. 2023 Sep 28;55:67. doi: 10.1186/s12711-023-00840-z (PMC10537099; doi:10.1186/s12711-023-00840-z)
Supplement: Supplementary file 2 — Additional file 2: Table S1. Estimation of genetic parameters, accuracy and bias of EBV from GLMM for all combinations of ${h}_{o}^{2}$ho2 and ${r}_{A}$rA. The table contains complete estimated genetic parameters, accuracy and bias for all of the set-ups. [file 12711_2023_840_MOESM2_ESM.docx]

**Additional file 2: Table S1. Estimation of genetic parameters, accuracy and bias of EBV from GLMM for all combination of** $\boldsymbol{h}_{\boldsymbol{o}}^{\boldsymbol{2}}$ **and** $\boldsymbol{r}_{\boldsymbol{A}}$**.**

|  |  | $r_{A}$ | | | | | |
| --- | --- | --- | --- | --- | --- | --- | --- |
| $h_{o}^{2}$ |  | -0.5 | | 0 | | 0.5 | |
|  |  | Estimated | True | Estimated | True | Estimated | True |
| 0.05 | $\mu_{\alpha}=\mu_{\beta}$ | -2.265 | -2.3 | -0.271 | -2.3 | -0.268 | -2.3 |
|  | $\sigma_{A_{\alpha}}^{2}$ | 0.0131 | 0.012 | 0.0121 | 0.012 | 0.0123 | 0.012 |
|  | $\sigma_{A_{\beta}}^{2}$ | 0.0110 | 0.012 | 0.0138 | 0.012 | 0.0119 | 0.012 |
|  | $\sigma_{{Ep}_{\alpha}}^{2}$ | 0.0112 | 0.012 | 0.0117 | 0.012 | 0.0110 | 0.012 |
|  | $\sigma_{{Ep}_{\beta}}^{2}$ | 0.0106 | 0.012 | 0.0113 | 0.012 | 0.0128 | 0.012 |
|  | $r_{A}\sigma_{A_{\alpha}}\sigma_{A_{\beta}}$ | -0.0053 | -0.006 | 0.0009 | 0 | 0.0056 | 0.006 |
|  | Accuracy | 0.455 (±0.009) | | 0.471 (±0.009) | | 0.448 (±0.008) | |
|  | Bias | 0.875 (±0.023) | | 0.847 (±0.027) | | 0.881 (±0.016) | |
|  |  | Estimated | True | Estimated | True | Estimated | True |
| 0.1 | $\mu_{\alpha}=\mu_{\beta}$ | -2.227 | -2.3 | -0.238 | -2.3 | -0.236 | -2.3 |
|  | $\sigma_{A_{\alpha}}^{2}$ | 0.0421 | 0.038 | 0.0396 | 0.038 | 0.0423 | 0.038 |
|  | $\sigma_{A_{\beta}}^{2}$ | 0.0396 | 0.038 | 0.0375 | 0.038 | 0.0410 | 0.038 |
|  | $\sigma_{{Ep}_{\alpha}}^{2}$ | 0.0367 | 0.038 | 0.0358 | 0.038 | 0.0374 | 0.038 |
|  | $\sigma_{{Ep}_{\beta}}^{2}$ | 0.0372 | 0.038 | 0.0361 | 0.038 | 0.0359 | 0.038 |
|  | $r_{A}\sigma_{A_{\alpha}}\sigma_{A_{\beta}}$ | -0.0181 | -0.019 | 0.0012 | 0 | 0.0176 | 0.019 |
|  | Accuracy | 0.571 (±0.008) | | 0.595 (±0.011) | | 0.563 (±0.015) | |
|  | Bias | 0.917 (±0.019) | | 0.911 (±0.022) | | 0.896 (±0.018) | |
|  |  | Estimated | True | Estimated | True | Estimated | True |
| 0.2 | $\mu_{\alpha}=\mu_{\beta}$ | -2.255 | -2.3 | -0.261 | -2.3 | -0.238 | -2.3 |
|  | $\sigma_{A_{\alpha}}^{2}$ | 0.1724 | 0.170 | 0.1719 | 0.170 | 0.1697 | 0.170 |
|  | $\sigma_{A_{\beta}}^{2}$ | 0.1703 | 0.170 | 0.1706 | 0.170 | 0.1723 | 0.170 |
|  | $\sigma_{{Ep}_{\alpha}}^{2}$ | 0.1652 | 0.170 | 0.1649 | 0.170 | 0.1689 | 0.170 |
|  | $\sigma_{{Ep}_{\beta}}^{2}$ | 0.1709 | 0.170 | 0.1655 | 0.170 | 0.1665 | 0.170 |
|  | $r_{A}\sigma_{A_{\alpha}}\sigma_{A_{\beta}}$ | -0.0853 | -0.085 | -0.0004 | 0 | 0.0796 | 0.085 |
|  | Accuracy | 0.668 (±0.012) | | 0.713 (±0.017) | | 0.659 (±0.010) | |
|  | Bias | 0.936 (±0.030) | | 0.925 (±0.019) | | 0.928 (±0.021) | |

Standard errors are in brackets. For the bias, regression coefficients of true breeding values on EBV are shown, so that values smaller than one indicate overestimation of breeding values. $h_{o}^{2}$ is the observed-scale heritability of the mean of 2500 binary observations with on average 25 successes, based on a linear mixed model.

The true values of variances were the simulate input values, which was also shown in Table 1.

$r_{A}\sigma_{A_{\alpha}}\sigma_{A_{\beta}}$ is the genetic covariance between the two trait. The covariance were estimated from GLMM. Since the genetic correlation $r_{A}=0$, true value of the covariance was zero as well.
