# Supplementary material for: Towards genetic improvement of social behaviours in livestock using large-scale sensor data: data simulation and genetic analysis
Source: Genet Sel Evol. 2023 Sep 28;55:67. doi: 10.1186/s12711-023-00840-z (PMC10537099; doi:10.1186/s12711-023-00840-z)
Supplement: Supplementary file 3 — Additional file 3: Text S2. Estimation of the upper bound of accuracy in the GLMM. The file shows the calculation of the upper bound of the accuracy one could get with ${\sigma }_{A}^{2}={\sigma }_{Ep}^{2}$σA2=σEp2. [file 12711_2023_840_MOESM3_ESM.docx]

**Additional file 3**

**Estimation of the upper bound of accuracy in the GLMM.**

Assuming a half-sib family structure was applied, in which: each sire was mated with (k+1) dam, and each dam produced (m+1) offspring. Thus each offspring had m full sibs and k(m+1)=n half-sibs. (in our simulation: k=3, m=4, n=15). Considering the following two cases:

*Case 1:*

$$Var\left( A \right)=Var\left( E_{P} \right)=0.005, Var\left( \varepsilon\right)=0.99$$

$$Var\left( P \right)=Var\left( A \right)+Var\left( E_{P} \right)+Var\left( \varepsilon\right)=1$$

*So*

$$h^{2}=\frac{Var\left( A \right)}{Var\left( P \right)}=0.005, r=\frac{Var\left( A \right)+Var\left( E_{P} \right)}{Var\left( P \right)}=0.01$$

Information used for BV estimation: $t$ own record, $\mathrm{mt}$ full-sibs, $\mathrm{nt}$ half-sibs

*Case 2:*

$$Var\left( A \right)=Var\left( E_{P} \right)=0.5, Var\left( \varepsilon\right)=0$$

$$Var\left( P \right)=Var\left( A \right)+Var\left( E_{P} \right)+Var\left( \varepsilon\right)=1$$

*So*

$$h^{2}=\frac{Var\left( A \right)}{Var\left( P \right)}=0.5, r=\frac{Var\left( A \right)+Var\left( E_{P} \right)}{Var\left( P \right)}=1$$

Information we use for BV estimation: $1$ own record, $m$ full-sibs, $n$ half-sibs

When $t\to\infty$, Case 1 and Case 2 are equivalent and has the same accuracy. That’s because when number of record is very large, $Var\left( \varepsilon\right)$ is canceled and does not play a role in accuracy. Our simulation was actually Case 1, with very low heritability and repeatability in the underlying level. However, we had millions of binary records per individual, so we have Case 1 with very big records per individual. Therefore we can simplified our model to Case 2.

Considering using selection index theory for approximate accuracy calculation.

$$I\left( Index \right)= b_{1}X_{1}+b_{2}X_{2}+b_{3}X_{3}$$

where

$$X_{1}=1 own record,$$

$$X_{2}=mean of m full-sib record (each sib has one own record)$$

$$X_{3}=mean of n half-sib record (each sib has one own record)$$

Therefore

$$Var\left( X_{1} \right)= Var\left( P \right)=1,$$

$$Cov\left( X_{1},X_{2} \right)=r_{full-sib}*h^{2}*Var\left( P \right)=0.5*0.5*1=0.25$$

$$Cov\left( X_{1},X_{3} \right)=r_{half-sib}*h^{2}*Var\left( P \right)=0.25*0.5*1=0.125$$

$$Var\left( X_{2} \right)=Var\left( \frac{x1+x2+\ldots+xm}{m} \right)= \frac{m*Var\left( P \right)+m\left( m-1 \right)r_{full-sib}*h^{2}*Var\left( P \right)}{m^{2}}=\frac{1+0.25\left( m-1 \right)}{m}=0.25+\frac{0.75}{m}$$

$$Cov\left( X_{2},X_{3} \right)=r_{half-sib}*h^{2}*Var\left( P \right)=0.25*0.5*1=0.125$$

$$Var\left( X_{3} \right)=Var\left( \frac{x1+x2+\ldots+xn}{n} \right)=\frac{nVar\left( P \right)+nm*r_{full-sib}*h^{2}*Var\left( P \right)+n\left( k-1 \right)\left( m+1 \right)*r_{half-sib}*h^{2}*Var\left( P \right)}{n^{2}}=\frac{1+0.25m+0.125\left( k-1 \right)\left( m+1 \right)}{n}=\frac{0.875+0.125m+0.125k+0.125km}{n}=0.125+\frac{0.125m}{n}+\frac{0.875}{n}$$

Thus the P matrix is :

$$\boldsymbol{P}=\left( \begin{matrix} Var\left( X_{1} \right) & Cov\left( X_{1},X_{2} \right) & Cov\left( X_{1},X_{3} \right) \\ Cov\left( X_{1},X_{2} \right) & Var\left( X_{2} \right) & Cov\left( X_{2},X_{3} \right) \\ Cov\left( X_{1},X_{3} \right) & Cov\left( X_{2},X_{3} \right) & Var\left( X_{3} \right) \end{matrix} \right)=\left( \begin{matrix} 1 & 0.25 & 0.125 \\ 0.25 & 0.25+\frac{0.75}{m} & 0.125 \\ 0.125 & 0.125 & 0.125+\frac{0.125m}{n}+\frac{0.875}{n} \end{matrix} \right)$$

and G matrix is:

$$\boldsymbol{G}=\left( \begin{matrix} Cov\left( X_{1},A_{1} \right) \\ Cov\left( X_{1},A_{1} \right) \\ Cov\left( X_{2},A_{1} \right) \end{matrix} \right)=\left( \begin{matrix} h^{2} \\ r_{full-sib}*h^{2} \\ r_{half-sib}*h^{2} \end{matrix} \right)Var\left( P \right)=\left( \begin{matrix} 0.5 \\ 0.25 \\ 0.125 \end{matrix} \right)$$

When *k=3, m=4, n=k(m+1)=15* (in our simulation):

$$\boldsymbol{P}=\left( \begin{matrix} 1 & 0.25 & 0.125 \\ 0.25 & 0.4375 & 0.125 \\ 0.125 & 0.125 & 0.2167 \end{matrix} \right), \boldsymbol{P}^{-1}=\left( \begin{matrix} 0.1887 & -0.5787 & -0.3519 \\ -0.5787 & 3.0186 & -1.4076 \\ -0.3519 & -1.4076 & 5.6305 \end{matrix} \right)$$

$$\boldsymbol{b}=\boldsymbol{P}^{-1}\boldsymbol{G}=\left( \begin{matrix} 0.4057 \\ 0.2893 \\ 0.1760 \end{matrix} \right)$$

$$Var\left( I \right)=\boldsymbol{b}^{'}\boldsymbol{Pb}=0.2972$$

$$accuracy=\sqrt{\frac{Var\left( I \right)}{Var\left( A \right)}}=\sqrt{\frac{0.2972}{0.5}}=0.7710$$

When k=infinite, m= infinite, n= infinite (very large family structure):

$$\boldsymbol{P}=\left( \begin{matrix} 1 & 0.25 & 0.125 \\ 0.25 & 0.25 & 0.125 \\ 0.125 & 0.125 & 0.125 \end{matrix} \right), \boldsymbol{P}^{-1}=\left( \begin{matrix} 1.3333 & -1.3333 & 0 \\ -1.3333 & 9.3333 & -8 \\ 0 & -8 & 16 \end{matrix} \right)$$

$$\boldsymbol{b}=\boldsymbol{P}^{-1}\boldsymbol{G}=\left( \begin{matrix} 0.3333 \\ 0.6667 \\ 0 \end{matrix} \right) (so half sibs donot play a role if you already have infinite full sibs.)$$

$$Var\left( I \right)=\boldsymbol{b}^{'}\boldsymbol{Pb}=0.3333$$

$$accuracy=\sqrt{\frac{Var\left( I \right)}{Var\left( A \right)}}=\sqrt{\frac{0.3333}{0.5}}=0.8164$$

In conclusion: (1) With the simulated family structure in this study (4 full-sibs + 15 half-sibs per individual), the highest accuracy one could ever get is 0.7710; (2)With infinite full sib and half sibs, the highest accuracy one could ever get is 0.8164.
